# Cyber-WISE: A Cyber-Physical Deep Wireless Indoor Positioning System and Digital Twin Approach

**DOI:** 10.3390/s23249903

**Published:** 2023-12-18

**Authors:** Muhammed Zahid Karakusak, Hasan Kivrak, Simon Watson, Mehmet Kemal Ozdemir

**Affiliations:** 1 Graduate School of Engineering and Natural Sciences, Istanbul Medipol University, 34810 Istanbul, Turkey; 2 Department of Electronics Technology, Karabuk University, 78010 Karabuk, Turkey; 3 Department of Computer and Information Sciences, Northumbria University, Newcastle upon Tyne NE1 8ST, UK; hasan.kivrak@northumbria.ac.uk; 4 Department of Electrical and Electronic Engineering, The University of Manchester, Manchester M13 9PL, UK; simon.watson@manchester.ac.uk; 5 Department of Computer Engineering, Istanbul Medipol University, 34810 Istanbul, Turkey; mkozdemir@medipol.edu.tr

**Keywords:** Internet of things (IoT), digital twins, cyber-physical systems (CPSs), smart space, indoor localization, wireless LAN positioning, fingerprint matrix, received signal strength (RSS), deep learning, long short-term memory (LSTM)

## Abstract

In recent decades, there have been significant research efforts focusing on wireless indoor localization systems, with fingerprinting techniques based on received signal strength leading the way. The majority of the suggested approaches require challenging and laborious Wi-Fi site surveys to construct a radio map, which is then utilized to match radio signatures with particular locations. In this paper, a novel next-generation cyber-physical wireless indoor positioning system is presented that addresses the challenges of fingerprinting techniques associated with data collection. The proposed approach not only facilitates an interactive digital representation that fosters informed decision-making through a digital twin interface but also ensures adaptability to new scenarios, scalability, and suitability for large environments and evolving conditions during the process of constructing the radio map. Additionally, it reduces the labor cost and laborious data collection process while helping to increase the efficiency of fingerprint-based positioning methods through accurate ground-truth data collection. This is also convenient for working in remote environments to improve human safety in locations where human access is limited or hazardous and to address issues related to radio map obsolescence. The feasibility of the cyber-physical system design is successfully verified and evaluated with real-world experiments in which a ground robot is utilized to obtain a radio map autonomously in real-time in a challenging environment through an informed decision process. With the proposed setup, the results demonstrate the success of RSSI-based indoor positioning using deep learning models, including MLP, LSTM Model 1, and LSTM Model 2, achieving an average localization error of ≤2.16 m in individual areas. Specifically, LSTM Model 2 achieves an average localization error as low as 1.55 m and 1.97 m with 83.33% and 81.05% of the errors within 2 m for individual and combined areas, respectively. These outcomes demonstrate that the proposed cyber-physical wireless indoor positioning approach, which is based on the application of dynamic Wi-Fi RSS surveying through human feedback using autonomous mobile robots, effectively leverages the precision of deep learning models, resulting in localization performance comparable to the literature. Furthermore, they highlight its potential for suitability for deployment in real-world scenarios and practical applicability.

## 1. Introduction

Indoor positioning systems are an essential part of location-aware applications and services in indoor environments, enabling localization, tracking, and monitoring of assets. These systems have diverse applications across various domains, including factory logistics, healthcare, asset tracking in office environments [1,2], medical equipment [3], and patients [4], as well as monitoring indoor activities and optimizing processes and resource allocations [5]. They also enable the delivery of location-based services, such as providing recommendations to tourists [6], assisting customers in navigating in indoor environments [7], and offering directions in shopping malls [8]. To meet these demands, extensive research has been conducted, utilizing a range of technologies such as visual [9], geometric [10,11], ultrasonic [12], and RF-based sensors [13,14].

The wireless local area network (WLAN) positioning method is one of the most commonly used RF-based techniques for indoor positioning. It relies on the characteristics of wireless signals, such as the angle of arrival (AOA), time of arrival (TOA), and received signal strength (RSS) based on the transmit-receive relationship of radio signals, without requiring connection to Wi-Fi networks [15]. However, modeling the relationship between RSS and position is challenging due to environmental constraints, including multipath interference or non-line-of-sight (NLOS) conditions. These factors can cause unpredictable signal propagation and random variations of RSS samples, even at fixed positions [16]. Furthermore, to tackle challenges like device height and heterogeneity, an approach involves manually adjusting RSS values for various testing devices using a linear transformation approach, incorporating diverse transformation functions such as time-space sampling [17], Kullback–Leibler divergence [18], and the Gaussian fit sensor model [19]. Because simple linear relationships may not effectively characterize the difference across mobile devices [18], researchers also introduce calibration-free methods where a new type of fingerprint is generated using absolute RSS values. For instance, RSS differences (signal strength difference) [20] and signal strength ratios [21] between pairs of access points (APs) serve as location fingerprints. Additionally, weighting schemes that consider the relationship of RSS values for each RSS are employed without extra calibration on test devices [22,23,24].

Fingerprint-based techniques [25,26] are a common approach for describing the relationship between RSS and position. These techniques rely on previously collected measurement data, particularly in multipath-rich fading indoor environments, as compared to distance-based trilateration and direction-based triangulation methods [27]. Therefore, accurate data collection is important for ensuring fingerprint-based techniques’ effectiveness and reliability to match radio signatures with specific locations. However, collecting data for fingerprint-based methods is a labor-intensive, time-consuming, and error-prone process, especially when working in large areas or scaling up systems [28].

The application of a smart space concept [29] to wireless indoor positioning can be applied to address the challenge of data collection by semi- or fully autonomously creating a radio map with digital twin (DT) integration. This approach can also overcome the scalability disadvantage of fingerprint-based systems [30,31] by providing a flexible and easily deployable system that can be integrated with existing network infrastructure. However, the implementation of a smart space setting involves cyber-physical systems (CPSs) infrastructure along with other requirements such as security, privacy, safety, and more [32]. CPSs are a new generation of integrated frameworks and mechanisms that enable the creation of living virtual DTs that replicate the behavior of their physical counterparts. These systems can improve efficiency, productivity, and collaborative working and enable the remote operation of complex physical systems [33]. This is facilitated through the combination of the Internet of Things (IoT) and twinning technologies, which not only provide simple synthetic representations but also enable synchronous bi-directional streaming of up-to-date data between the physical and digital spaces [34].

Considering cyber-physically in indoor positioning can also improve the efficiency and controllability of complex systems through intelligent human-machine interaction in cyberspace through a user interface [32]. Human operators can utilize DTs to remotely observe the real-time conditions in the operating environment and make better decisions. This capability can be particularly useful for identifying potential problems or opportunities for improvement, guiding better planning of the physical space, APs, and/or reference point (RP) layout decisions [35].

### Contribution

This paper delivers new insights into wireless indoor positioning systems by approaching them from a cyber-physical perspective, focusing on fingerprinting techniques associated with data collection. This new proposed concept addresses challenging and laborious Wi-Fi site surveys to construct radio maps for a variety of indoor environments, such as modern smart buildings or digital workplaces, as well as challenging and demanding environments like nuclear facilities. In this regard, the research presents an innovative concept in wireless indoor positioning systems by adopting a cyber-physical approach with the following major contributions:

- We propose a cyber-physical wireless indoor positioning system framework to dynamically address the labor-intensive, time-consuming, error-prone construction of radio maps. This framework allows for regular radio map reconstruction and scalability of fingerprint-based positioning methods through dynamic Wi-Fi RSS surveying using (semi) autonomous mobile data collectors via digital twins.
- We develop a live synthetic 3D digital twin environment and user-friendly graphical user interface that incorporate human feedback in multiple ways, including the determination of reference point placement for fingerprint layout, facilitating safety-critical and remote missions, teleoperation of robots, and monitoring mission states and robot operations.
- We validate the feasibility of the proposed framework in a representative real-world nuclear environment and demonstrate the positioning performance of our framework using various deep learning algorithms.

## 2. Related Work

IoT and DT solutions are increasingly being employed to address indoor positioning challenges. This trend is expected to continue in the foreseeable future, as evidenced by a growing number of publication trends on IoT-based applications for indoor positioning, including review papers [36,37] and research articles (as shown in Table 1). Moreover, commercial DT services offered to various sectors have emerged alongside recent research activities [38,39,40,41,42].

Furthermore, research in the area of IoT and DT-enabled indoor positioning has pursued several avenues. Early work was primarily focused on the potential usefulness of the IoT concept for indoor positioning, rather than its practical applications. While some papers, such as [43,44,45], mentioned Industry 4.0 and IoT in their title or abstract, they did not discuss the development or practical implementation of these concepts. Table 1 presents a review of research articles that focus on implementing indoor positioning through IoT or DT infrastructure at different levels. Each paper is assessed and grouped by year, use case, the technology used, signal characteristics, indoor positioning techniques, context, interactivity, and application area. Additionally, each of them is evaluated based on different maturity levels of DT. These maturity levels include hierarchical naming conventions for data twin, asset twin, predictive twin, interactive twin, and cognitive twin [46,47].

As an example of IoT-based indoor positioning, Ref. [6] developed an indoor tracking system for tourism applications that tracks visitor activities and gathers information about their behavior and interests, such as the order in which they visit points of interest and the level of interest they exhibit for each. The system uses range-based trilateration and triangulation positioning techniques based on Bluetooth low energy (BLE)-based received signal strength indication (RSSI) to determine the level of proximity to each interest point rather than the precise location of a person. Although the study did not focus on interface or visualization, it did capture useful real-time analytics for each visitor and provided a tour report. Therefore, this study can be considered an example of a data twin in terms of the various maturity levels of DTs.

**Table 1:** A review of indoor localization techniques in the literature through IoT and DT deployment.

Ref. ID	Year	Use-Case	Technology	Metric	Technique	Method	Context	Interaction	App.**Area
[48]	2017	Estimating region of interest	BLE	RSS	Localization	Fingerprint based	IoT	✗	Campus
[49]	2020	Analysis of visitor routes	BLE	RSS	Proximity and localization	Range based	IoT	✗	Museum
[6]	2021	Analysis of visitor routes	BLE	RSS	Proximity	Range based	IoT	✗	Tourism
[5]	2021	Asset tracking	BLE	RSS	Tracking	Range based	DT	✗	Industry and logistics
[1]	2021	Asset tracking	BLE	RSS	Localization	Fingerprint based	DT	✗	Industry and logistics
[2]	2022	Asset tracking	BLE and UWB	RSS	Tracking	Fingerprint based	IoT and DT	✗	Industry and logistics
[35]	2022	Occupancy and radio propagation analysis	UWB	TOA	Proximity and localization	Fingerprint based	DT	✓	Rectangular open area
Proposed	2023	Occupancy and radio propagation analysis	Wi-Fi	RSS	Tracking	Fingerprint based	CPS	✓	Rep.* nuclear environment

* Representative, ** Application

Several researchers have focused on visualizing indoor positioning data and performing data analytics to enhance the traceability and visibility of physical objects, resulting in what is known as an asset twin. For instance, in Ref. [48], an IoT-based design is developed to identify locations within indoor environments. The design includes a dashboard interface, which allows users to gain insights into historical perceived data and estimated location results. In the study, two different fingerprint-based k-nearest neighbors (KNN) and K-means clustering classification algorithms are implemented using BLE-based RSSI.

Similarly, in Ref. [2], the implementation of industrial IoT and DT technology is introduced to facilitate the inspection, maintenance, and packaging processes of finished goods within a workshop setting. The study proposed a set of DT services, including a dynamic map, overtime alarm, and mobile application, to improve operational efficiency and enhance information traceability and visibility of physical objects. Moreover, the study proposed a long short-term memory (LSTM)-based indoor positioning system that uses both ultra-wideband (UWB) and BLE technologies to leverage the RSSI signal characteristics for real-time tracking of finished goods.

Furthermore, in Ref. [5], a comprehensive tool designed to support the integration of IoT and DT with indoor positioning systems is demonstrated. The study addresses communication technology, localization techniques, hardware components, and application design. The researchers developed an attendance management system that uses a BLE-based indoor positioning system, utilizing RSSI in the path loss model to track employees’ attendance and monitor their presence. The concept involves using employees’ smartphones as identification markers with an Android application.

However, aside from asset twins, few studies have investigated utilizing predictive or interactive capabilities to enhance recommendations for decision-making in indoor positioning applications by incorporating dynamic and recurring data reception. For instance, Ref. [49] developed an IoT-based indoor localization system to improve the user experience in a museum. The system provides valuable information about exhibits or collections by notifying nearby visitors through an Android application. It also collects useful analytics of each visit and offers recommendations to users based on their historical data. The trilateration method using BLE-based RSSI information, besides the Kalman filter, is implemented as a positioning algorithm. In terms of providing recommendations in addition to the user interface, this study may serve as an example of the next stage of DT, known as predictive twins.

Likewise, another study [1] proposes a DT-enabled framework for real-time indoor human safety tracking in airport cargo terminals. The framework visualizes and monitors the health status and location information of humans on a dashboard, predicting abnormal motionless behavior remotely for safety management. An algorithm for detecting abnormal motionless behavior and self-learning genetic positioning is developed using BLE-based RSSI in real-time. While these applications are successful in their respective fields, they are not yet true DTs because they lack bi-directional interaction or feedback to guide physical space decision-making.

An example of a bi-directional DT application with a feedback scheme is given in Ref. [35]. The study uses the propagation characteristics of UWB signals to analyze occupancy in a building with the help of DTs and then guides AP layout decision-making accordingly. This two-way communication enhances the planning of physical spaces, reduces NLOS situations, and results in improved positioning accuracy and increased value of DTs. The study also develops a fingerprint-based neural network positioning algorithm using the TOA signal characteristic of UWB. While this study is the first interactive and mature DT in the field of indoor positioning that we found, it is not clear from the paper how signal propagation or digitalization of occupancy analysis effectively aids AP location or DT visualization.

To summarize, the progression of DTs across the various stages has already begun for indoor positioning systems. Table 2 presents an assessment of the maturity of the current state of studies in terms of DT deployment. The stages progress from data twin to cognitive twin, meaning they advance from those that display only the current condition of an asset to those incorporating technologies for autonomous and adaptive self-enhancements in changing conditions. To the best of the authors’ knowledge, there have been no previous instances of cognitive twin development for indoor positioning systems. This study aims to fill this gap by proposing the development of cognitive twin capabilities. Specifically, it (1) employs autonomous Wi-Fi RSS surveying to dynamically construct radio and heat maps using mobile data collectors (e.g., robot(s)) with human-in-the-loop control through a user interface. This facilitates adaptation to new scenarios, changes in the environment, or AP locations. It will also make data collection easier to scale up and increase the efficiency of fingerprint-based positioning methods. Additionally, this study (2) develops a graphical user interface for visualizing, interacting with, and monitoring the cyber-physical indoor environment to minimize the need for human intervention and enable remote applications.

For the rest of this paper, Section 3 describes the design methodology, defines the components of the cyber-physical wireless indoor positioning system, and provides an overview of the overall integration. In Section 4, the whole system is evaluated in an experimental setup, and the results are presented, while Section 5 concludes the study with a discussion.

## 3. Cyber-Physical Wireless Indoor Positioning System

This section presents the CPS infrastructure design methodology, an approach that tackles the operational and planning decision challenges associated with indoor positioning problems.

### 3.1. Problem Formulation

Existing fingerprint-based indoor positioning systems face several challenges and limitations. For instance, they often lack scalability, meaning that they are not easily adaptable to different environments and may not work effectively in large evolving spaces such as office buildings, shopping malls, airports, and hospitals. Furthermore, the reliance on human presence in these systems can result in increased labor costs, time-consuming processes, error-prone outcomes, and safety concerns, especially in extreme environments like nuclear facilities, where human access may be restricted or hazardous. Additionally, these systems lack situational awareness, which can hinder people’s ability to make informed decisions. In this regard, the following problem statements are defined.

1. **Scalability**. The system should be adaptable and applicable to diverse indoor settings as well as challenging remote environments.
2. **Situational awareness**. The system should augment situational awareness and support humans in making better decisions.
3. **Minimize the reliance on human presence**. The system should minimize human presence in the field to both lower the costs of labor related to data collection and improve the safety of personnel and facilities.
4. **Precise and efficient data collection and model**. The system should provide precise ground-truth data collection for accurate positioning methods.
5. **Efficient communication network.** The system should provide acceptable latency in updates to provide timely and actionable information for seamlessly integrating physical and digital spaces.

### 3.2. System Requirements

**Figure 1:**
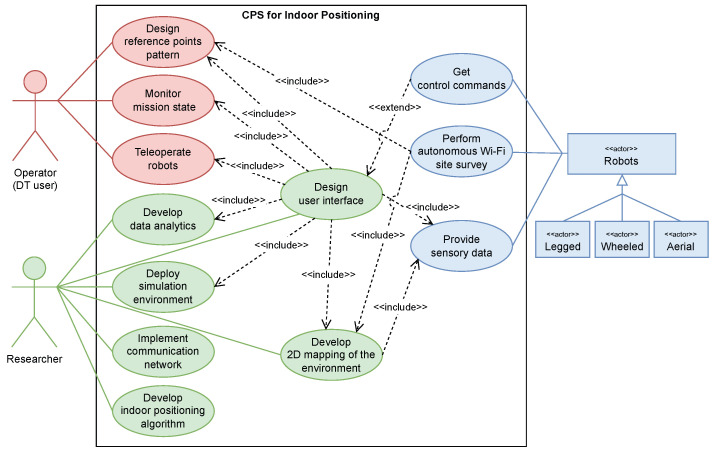
A UML use-case diagram of the proposed system requirements.

A UML use-case diagram, given in Figure 1, identifies system requirements of problem formulation through various use cases and interactions coordinated by various types of actors. The system involves three main actors: the human operator (DT user), the researcher(s), and the mobile robot(s). In the development of a cyber-physical indoor positioning architecture, the initial step comprises identifying the robot types, their sensors, and behaviors for the mission environment. This includes essential functionalities such as control, mapping, navigation, and other foundational capabilities like conducting autonomous Wi-Fi site surveys, receiving motor commands from teleoperation data sources, providing sensory data for visualization, and generating 2D mapping. Subsequently, the deployment of a digital twin environment by researchers ensures that real-world assets are accurately represented and timely updated through communication networks, along with the design of user interface and the development of data analytics. Finally, the integration of DT and its GUI enables oversight of mission states and operations of the robot(s), allowing human operator (DT user) intervention such as teleoperation of robots and determining reference points placement for fingerprint layout.

Recognizing the problem statements and system requirements emphasizes the need for a holistic, advanced concept or solution that seamlessly integrates indoor positioning and digital twinning technologies. In this context, a potential solution to overcome these limitations involves developing a CPS architecture that integrates indoor positioning and digital twin technologies.

**Figure 2:**
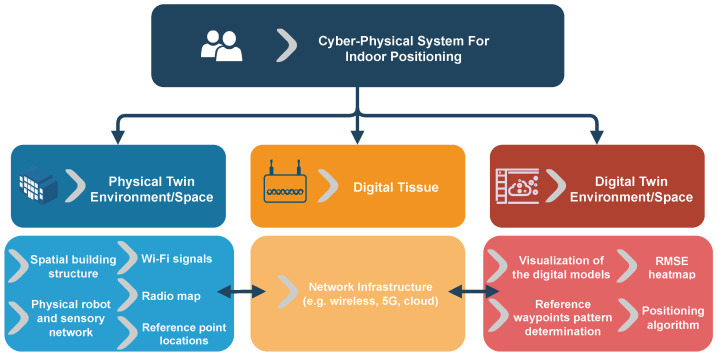
Diagrammatic visual conceptual model and components of the cyber-physical wireless indoor positioning system, along with corresponding assets at the bottom level.

Figure 2 illustrates the proposed components of the cyber-physical wireless indoor positioning system, which involves three major elements: (1) constructing a physical space or twin, (2) developing a DT, and (3) establishing a digital tissue or digital communication network. Each of these components will be discussed in detail in the subsequent subsections.

#### 3.2.1. Physical Twin

**Figure 3:**
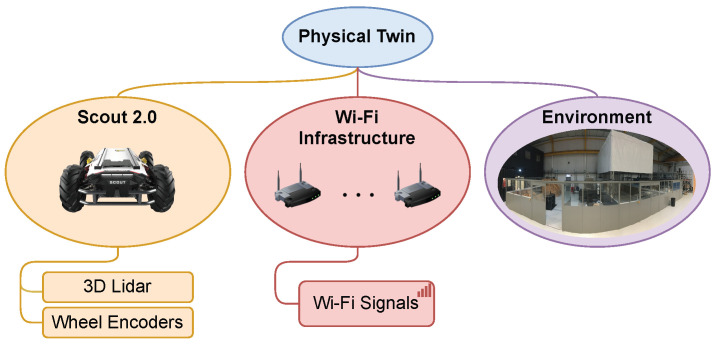
Elements of physical twin.

**Figure 4:**
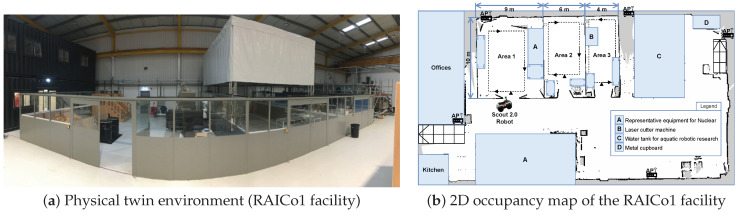
RAICo1 physical real-world environment and corresponding 2D map.

The physical twin is the real-world physical environment, including the spatial building structure, physical instances of robots, sensors, and a sensory network that includes APs. The proposed physical twin is depicted in Figure 3. It consists of the physical environment of the RAICo1 facility in West Cumbria, UK, depicted in Figure 4, which spans approximately 20m×40m. The facility is divided into three separate, fenced areas, each equipped with representative equipment and tools. Furthermore, an Agilex Scout 2.0 four-wheeled, differential-drive ground robot is employed, which is equipped with encoders on its wheels and a 3D laser capable of perceiving its surroundings with a range of 100 m and a sampling rate of 1875 @ 10 Hz. In addition, the existing wireless infrastructure of the facility is utilized for Wi-Fi RSS measurement from the APs. In summary, the physical twin contains valuable information about the physical environment, such as Wi-Fi signals, a radio map, and RP locations, which are accessible through the robotic platform.

#### 3.2.2. Digital Twin

A DT is a virtual replica or representation of a physical system that is synchronized with real-world assets and updated in near real-time [50]. When applied in the field of indoor positioning, DTs can lead to more efficient, scalable, and adaptable positioning systems by digitally representing physical systems and enabling interaction with humans. In this study, a 3D DT is developed by digitally modeling the physical environment and robots in a virtual or simulation environment to monitor the status of the robot while planning and executing its mission.

Furthermore, a graphical user interface (GUI) is integrated to facilitate interaction between users and the DT. This interactive interface assists in teleoperating the physical twin element of a mobile robot while simultaneously guiding the robot for a radio map and ground-truth locations, which are essential for developing fingerprinting-based techniques. This process involves making RP layout decisions based on the generated 2D dynamic occupancy map of the environment using the proposed interactive Wi-Fi site survey, which is detailed in the subsequent subsections.

#### 3.2.3. DT and Graphical User Interface

**Figure 5:**
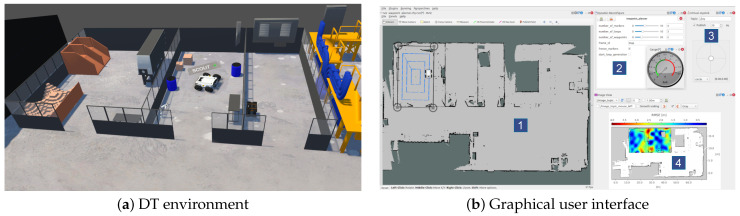
The DT environment together with the user interface.

The DT environment is developed based on the 3D building plan of the RAICo1 facility and the Agilex Scout 2.0 mobile robot shown in Figure 4a. Figure 5 presents an overview of the DT environment and GUI based on robot operating system (ROS) rqt [51]. The GUI provides multiple panels that support interactive Wi-Fi surveys and display live visualizations for analyzing and gaining insights into the robot’s actions and the overall system. Specifically, the GUI comprises the following windows:

1. Live mission monitoring and visualization window, which displays data collected from the physical twin.
2. RP generation parameter reconfiguration window.
3. Remote teleoperation window, which includes a virtual joystick and speedometer.
4. Positioning RMSE heat map window.

These windows allow for overseeing and coordinating the Wi-Fi site survey mission, as well as teleoperating the robot if necessary.

#### 3.2.4. Interactive and Autonomous Wi-Fi Site Survey

Various methods have been proposed for conducting wireless site coverage surveys, including passive, active, and predictive approaches [52]. In this study, a passive survey approach is employed to identify and characterize the signal propagation of the existing Wi-Fi infrastructure in the environment. The survey process is managed by a human-in-the-loop using a GUI and autonomously executed by a mobile robot. This approach enables the construction of highly adaptable radio maps for new scenarios or changes in environmental conditions or AP locations, thereby overcoming the limitations of scalability and laborious data collection typically associated with fingerprint-based methods.

**Figure 6:**
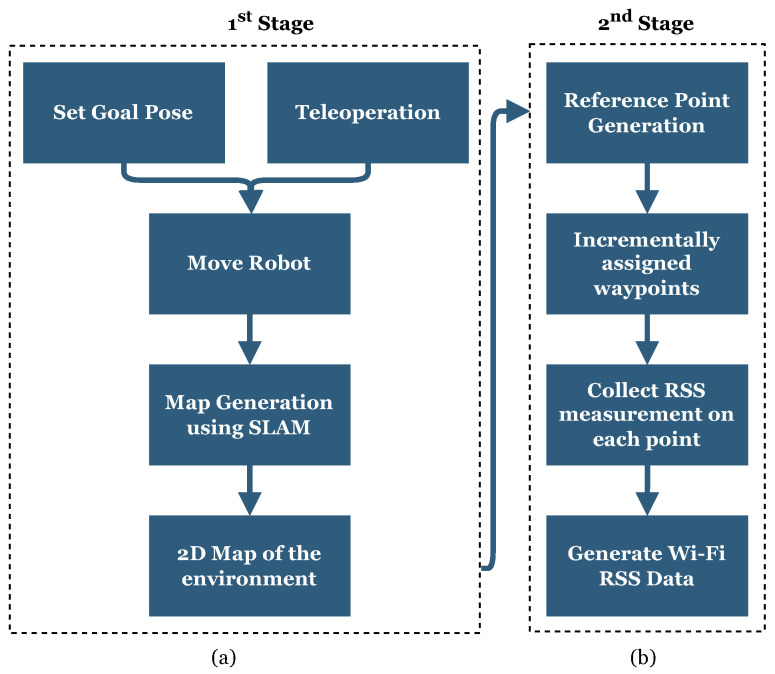
Flowcharts of interactive Wi-Fi site survey steps: (**a**) 1st Stage: Generating the map of the environment; (**b**) 2nd Stage: Generating RSS data on designated waypoints on the generated map autonomously.

Figure 6 illustrates the stages involved in the interactive Wi-Fi site survey. The initial step includes digitally constructing the 2D spatial geometry of the area by exploring and perceiving the indoor environment. In this stage, a robot equipped with a laser sensor employs the simultaneous localization and mapping (SLAM) algorithm to scan and map the unknown environment. The robot can be either remotely teleoperated or directed to specific poses within the GUI.

**Figure 7:**
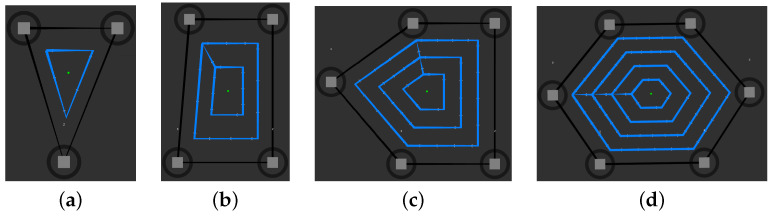
Examples of interactive reference point generation for Wi-Fi site surveys are presented in various shapes and designs. (**a**) A triangle shape with three markers, one inner loop, and one reference point between two vertices. (**b**) A rectangular shape with four markers, two inner loops, and two reference points between two vertices. (**c**) A pentagon shape with five markers, three inner loops, and three reference points between two vertices. (**d**) A hexagon shape with six markers, four inner loops, and four reference points between two vertices.

Once the mapping process is completed, the second stage involves distributing RPs throughout the mapped area using the RP generation tool [53]. This tool utilizes a dynamically adjusted marker pattern on a 2D grid map within the GUI, as shown in Figure 7. The RP generation tool provides adjustable dynamic parameters through the interface, including:

- The number of interactive outer markers to draw the boundary for surveying.
- The number of loops inside the outer boundary.
- The number or distance of RPs between two vertices for each loop.

Depending on environmental factors and design knowledge, the determination of the number of markers used to shape the outer boundary, the number of inner loops, and the number of RPs is facilitated through a human-in-the-loop decision-making process guided by the DT. After determining the number and placement of RPs through the GUI, these RPs are incrementally assigned as waypoints to the robot for measuring RSS values. The robot navigates these RPs and collects RSS measurements at each point. Ultimately, this results in obtaining a Wi-Fi RSS radio map of the environment along the traveled routes in the designated waypoints.

#### 3.2.5. Indoor Positioning Algorithm

**Figure 8:**
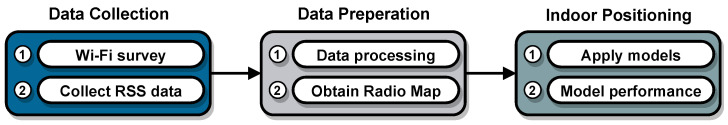
Overview of RSS-based WLAN positioning.

Figure 8 presents the three main steps of a WLAN positioning system. The first step involves storing RSS-position values obtained during the Wi-Fi site survey, which are later used as separate training, validation, and test data sets. The next step is to process and prepare the data as a radio map for input into the positioning models. The final step of WLAN positioning involves training models and estimating the 2D spatial positions for the tracking problem.

Deep learning methods are used to learn a model of the RSS-position relationship in order to accurately position a moving agent [54,55]. The methods utilized in this study are based on two deep learning techniques, namely multi-layer perceptron (MLP) and LSTM. These methods have been demonstrated to effectively tackle the Wi-Fi positioning problem as a regression task [55], with the root mean square error (RMSE) loss function (L) calculated by using Equation (1):

(1) $$L=\sqrt{E{\left[{\hat{x}}_{i}-y_{i}\right]}^{2}}$$

where ${\hat{x}}_{i}$ is the estimation for ith RP, and $y_{i}$ is the ground-truth position for ith RP.

Two different LSTM models are used: Model 1 takes as input a sequence of temporally consecutive lagged RSS observations. Given a time step *t* and the lag size *l*, this is represented as given in Equation (2).

(2) $${\mathrm{RSS}}_{\mathrm{seq}}=\Phi_{\mathrm{Model}\;1}(\left[{\mathrm{RSS}}_{t-l},\ldots ,{\mathrm{RSS}}_{t-1},{\mathrm{RSS}}_{t}\right];\theta_{l})$$

$\Phi_{\mathrm{Model}\;1}\left(\cdot ;\theta_{l}\right)$ is the packing function with lag size parameter $\theta_{l}$ which transforms the sequence of RSS observations, ${\mathrm{RSS}}_{t}$, into multiple samples, where each sample has a specified number of time steps, *l*, and is reshaped into three dimensions to match the input dimensions expected by the LSTM model, as given in Equation (3):

(3) $$\begin{array}{cc} {\mathrm{LSTM}}_{\mathrm{input}} & =\left[{\mathrm{RSS}}_{\mathrm{seq}}\right]\Rightarrow \left[\mathrm{samples}=n,\mathrm{timesteps}=l,\mathrm{features}=d\right] \end{array}$$

Likewise, Model 2 accepts lagged RSS observations along with the predicted position, ${\hat{p}}_{t-1}$, from the previous time step as input, as given in Equation (4).

(4) $${\mathrm{RSS}}_{\mathrm{seq}}=\Phi_{\mathrm{Model}\;2}(\left[{\mathrm{RSS}}_{t-l},\ldots ,{\mathrm{RSS}}_{t-1},{\mathrm{RSS}}_{t},{\hat{p}}_{t-1}\right];\theta_{l})$$

The deep network models used in this study are presented in detail in Ref. [55]. Consequently, deep wireless LAN positioning is formulated as a regression problem, and the performance of the model is evaluated based on the RMSE positioning error (Equation (12)).

#### 3.2.6. Digital Tissue

**Figure 9:**
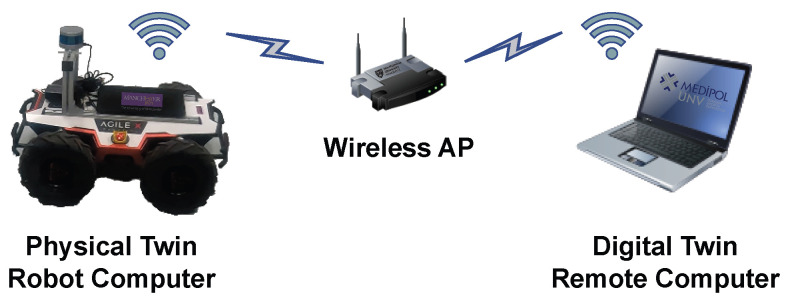
Wi-Fi communication infrastructure.

Digital tissue is another essential component of CPS and refers to the communication network that enables data to be transferred between digital and physical twins. It facilitates real (or right)-time and resilient sharing of data. The digital tissue framework also manages system overload caused by excessive data through bandwidth management and/or smart utilization of shared data. As for communication across pairs, Figure 9 shows the communication infrastructure of the ROS Wi-Fi network [56]. The bandwidth consumption of the entire system was measured at approximately 5.281 MB/s, equivalent to 42.253 Mbps, which is lower than the available bandwidth estimated at around 60 Mbps during the experimental setup, as shown in Table 3. Therefore, no further techniques for bandwidth management or smart data management processes are considered.

### 3.3. Overall Integration

**Figure 10:**
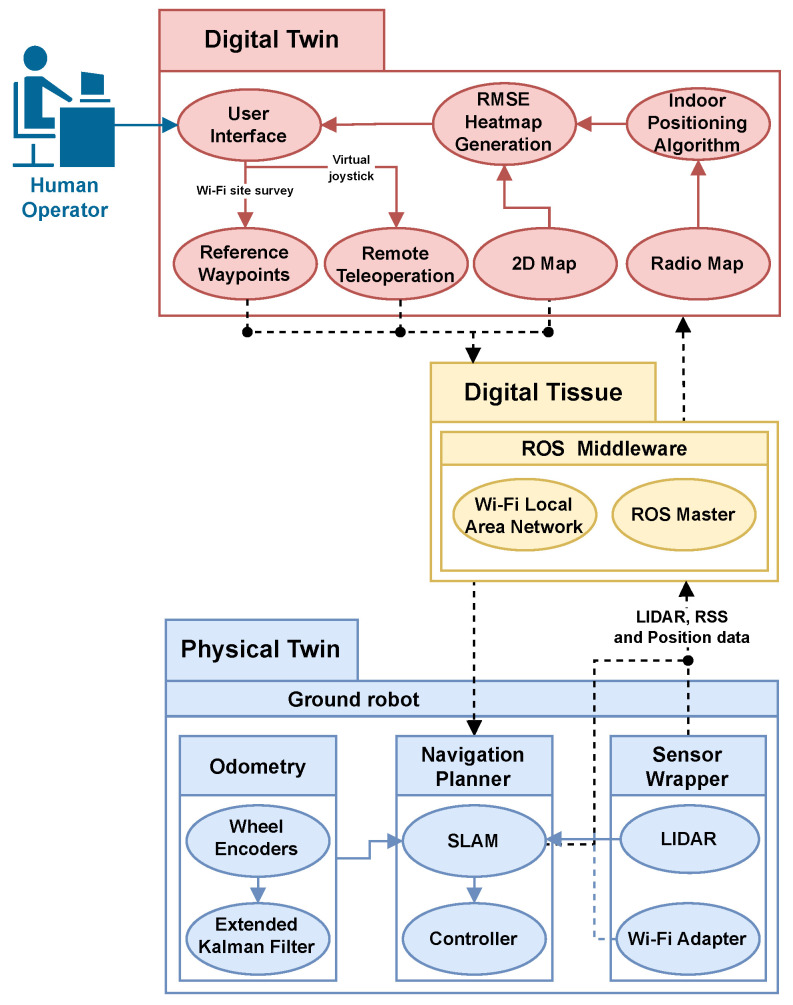
Proposed cyber-physical system design implementation and data flow diagram.

In the preceding subsections, the main elements of the cyber-physical indoor positioning system were discussed, which were categorized into physical twins, DTs, and digital tissue components. Figure 10 presents an overview of the proposed component implementations, illustrating how data flows through the components and connects to the human operator.

The physical robot instance provides ground-truth position data, which is estimated using the Grid Mapping SLAM algorithm [57] with incoming laser scans. In addition, the physical twin provides LIDAR and Wi-Fi RSS data through the robot’s sensors. The RSS-position data sent from the physical twin is stored and used to create a radio map. By performing 2D interpolation on positioning errors, a continuous RMSE heat map is generated, which is displayed on a 2D map to visualize the performance of the Wi-Fi positioning system.

On the other hand, the DT user interface serves as the primary point of contact between human operators and the physical assets, executing the live teleoperation and displaying the current state of the physical robot. Furthermore, the RP pattern planner tool allows operators to provide commands for desired measurement points on the map. These points are assigned as waypoints or routes for the physical twin to navigate and take RSS measurements. This tool facilitates more complex and demanding applications by providing easy customization of survey behavior.

To ensure the bi-directional operability of the CPS, the ROS Middleware Wi-Fi network architecture is utilized. This architecture enables data reception and transmission between the physical twin and the DT.

## 4. Experimental Setup and Results

The calculation of RSS, as discussed in Ref. [16], presents a significant challenge due to the dynamic nature of RSS in uncertain environments with spatio-temporal constraints. Therefore, it can be challenging to fully characterize and apply the relationship between RSS and position using theoretical or simulation radio propagation models, such as the commonly used path loss and shadowing model described in Equation (5) [58] to general environments. To address this limitation, the proposed CPS approach is evaluated based on experimental RSS data collected from real environments in a DT case scenario. When experimental RSS data are unavailable, the received power formulation in Equation (5) includes γ, which represents the path loss exponent describing signal attenuation with distance, and ϕ, which accounts for additional losses or gains. Typically, both γ and ϕ are usually determined through experiments.

(5) $$P_{r}\left(dB\right)=P_{t}\left(dB\right)+10\log_{10}K-10\gamma \log_{10}\left(\frac{d}{d_{0}}\right)-\phi \left(dB\right)$$

### 4.1. Data Collection

Experimental data sets for this study are collected at the RAICo1 facility, which houses fenced areas designated as Area 1, Area 2, and Area 3, as well as office areas and various test fields (Figure 4). The study relies on the existing WLAN communication infrastructure within the building, which orders the fixed number and placement of APs throughout the facility.

Data collection involves performing a current scan of all visible APs in the vicinity at each survey location. RSS measurements are collected using a laptop equipped with an Intel(R) Core(TM) i7-10750H Hex core processor, an Intel AX201/ Killer Wireless 1650 2 × 2 AC adapter, and an Ubuntu 20.04 operating system placed on the robot. RSS measurements are obtained through a publicly available Wi-Fi scan package [59], providing RSS measurements from Channels 1, 6, and 11 on the 2.4 GHz band with a sampling rate of approximately 1 sample/s, in line with a similar setup described in the existing literature [16]. During each survey location, RSS measurements are expressed as integers in decibel-milliwatt (dBm) on a scale ranging from 0 (indicating the maximum achievable signal strength relative to 1 milliwatt) to −100 (representing an unusable signal).

**Figure 11:**
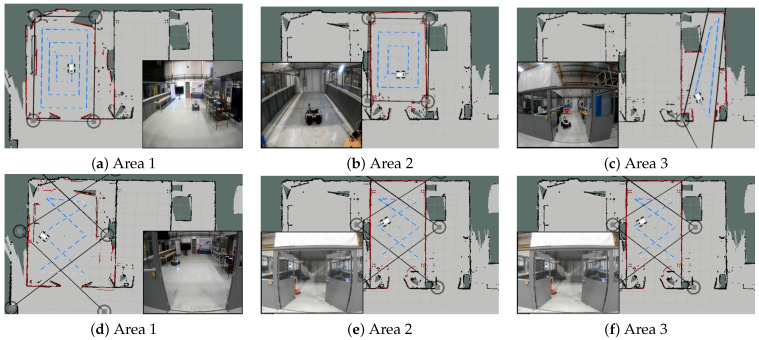
Map of the experimental areas and designated measurement points visualized with white arrows indicating the position and orientation of reference points during both the training phases (**a**–**c**) and the testing phases (**d**–**f**).

The number and placement of RPs are determined individually for each area, both for the training and test phases, through an interactive RP generator. Before starting the data collection process, the outer markers and waypoints are frozen to prevent human intervention. The layout and locations of RPs for training and test data sets are shown in Figure 11. These RPs are positioned to account for the presence of walls and other obstructions that may prevent navigating in certain areas. The spacing between RPs is set at 0.9 m, and the number of RPs is distributed accordingly [55,60].

During the training phase, the mobile robot remains stationary at each RP for approximately 10 s to collect n=10 RSS measurements from *d* detectable APs with a laptop placed on the mobile robot. The locations of RPs $p_{i}$ were generated using the SLAM algorithm. Therefore, the fingerprint matrix $F(p_{i})$ can be mathematically formulated considering *d* APs with *n* number of RSS samples received at the *i*th RP, as shown in Equation (6).

(6) $$F_{\mathrm{raw}}=F\left(p_{i}\right)=\left[\begin{array}{cccc} rss_{1}^{1} & rss_{1}^{2} & \cdots & rss_{1}^{d}\\ \vdots & \vdots & \ddots & \vdots \\ rss_{n}^{1} & rss_{n}^{2} & \cdots & rss_{n}^{d} \end{array}\right],p_{i}={\left[\begin{array}{cc} p_{x} & p_{y} \end{array}\right]}^{T}$$

In the case of collecting test data, a sequential traversal of a route of test points is performed, reflecting the tracking scenario. At each point, while moving, an RSS sampling is measured with a sample rate of 1 sample/s. Table 4 presents the number of train and test points per area, along with the RP planner parameters (Figure 7) and the observed number of APs. This set is used to evaluate the performance of the positioning methods used.

### 4.2. Data Preparation

Indoor environments often encounter issues such as multipath fading, shadow fading, and path loss, which are common characteristics of wireless propagation. Multipath fading happens when there are multiple paths through which the signals travel, resulting from transmission, reflection, and diffraction off various surfaces [61]. Moreover, shadow fading and path loss conditions can occur due to changes in the environment or the movement of people. These channel impediments vary over time, which means that the radio map used during the training phase and the RSS values measured during the test phase (which is a different time frame than training) of the positioning algorithm may not be consistent. These inconsistencies can lead to a degradation in positioning accuracy because the position estimators rely on the radio map to develop their perception of the environment.

To mitigate the negative impact of environmental uncertainties, three consecutive steps denoted as S are taken, as shown in Equation (7):

(7) $$S=[\Phi_{\mathrm{outlier}},\Phi_{\mathrm{missing}},\Phi_{\mathrm{selection}}]$$

The first step is outlier threshold filtering, where $\Phi_{\mathrm{outlier}}\left(\cdot ;\theta_{\mathrm{outlier}}\right)$ is applied to the measurement samples taken from the same point to account for natural variations in RSS data. This step can be represented, as given in Equation (8):

(8) $$F_{\mathrm{outlier}}=\Phi_{\mathrm{outlier}}\left(F_{\mathrm{raw}};\theta_{outlier}\right)$$

where $F_{\mathrm{outlier}}\in R^{n\times d}$ represents the filtered samples with *n* RSS measurements and *d* detectable APs. A defined threshold, $\theta_{outlier}$, is used to identify outlier samples, which are then corrected by replacing them with the average of the samples collected at each RP from each AP.

Secondly, considering that the set of visible APs can change over time, in the event of no data reception at a particular AP, the missing values are filled with the average RSS values, $\theta_{\mathrm{missing}}$, from that sequence. This process is denoted as $\Phi_{\mathrm{missing}}\left(\cdot ;\theta_{\mathrm{missing}}\right)$ and can be expressed as in Equation (9):

(9) $$F_{\mathrm{missing}}=\Phi_{\mathrm{missing}}\left(F_{\mathrm{outlier}};\theta_{\mathrm{missing}}\right)$$

Lastly, the AP selection step, denoted as $\Phi_{\mathrm{selection}}\left(\cdot ;\theta_{selection}\right)$, is performed based on the statistics of the RSS sample distribution. The aim is to exclude a number (*k*) of APs that do not contribute significantly. These APs are identified by their mean RSS value, which is approximately −100 dBm, along with a low standard deviation represented by $\theta_{\mathrm{selection}}$. As a result, the representation $F_{\mathrm{final}}\in R^{n\times (d-k)}$ is obtained in Equation (10), where *n* corresponds to the number of RSS measurements and (d−k) represents the remaining APs after excluding the non-significant ones.

(10) $$F_{\mathrm{final}}=\Phi_{\mathrm{selection}}\left(F_{\mathrm{missing}};\theta_{\mathrm{selection}}\right)$$

Eventually, a radio map R is obtained from RPs, $p_{i}$ across the area, as given in Equation (11):

(11) $$R=\{\left(p_{i},F_{\mathrm{final}}\left(p_{i}\right)\right)\;|\;i=1,\ldots ,N\}$$

The resulting radio map serves as the input for training deep learning-based positioning models. As a result, two separate data sets (training and testing) are created, where 23% of the training data is reserved as the validation data set, which will be used for parameter tuning.

### 4.3. Experimental Evaluation

One of the commonly used regression metrics, such as mean square error and mean absolute error, in indoor positioning algorithms to evaluate the performance of deep learning models is RMSE. While the RMSE loss function, as shown in Equation (1), is used during the training phase of a machine learning model, the positioning error is evaluated using the RMSE performance criteria, as indicated in Equation (12), on a test dataset. It quantifies the standard deviation of prediction errors, which shares the same unit of measurement as the actual positions, thus facilitating easier interpretation. This allows for a direct comparison of the error magnitude with the actual position unit, which is reported in meters.

(12) $$\mathrm{RMSE}≜\sqrt{\frac{1}{N_{p}}\sum_{n=1}^{N_{p}}{\left|p_{i}-{\hat{p}}_{i}\right|}^{2}}$$

### 4.4. Hyperparameter Optimization (HPO)

The process of tuning hyperparameter values, as shown in Table 5, is crucial for achieving efficient and accurate deep learning models. This process is often empirical and relies on the specific characteristics of the input dataset. Cross-validated grid search is employed to identify the best-performing hyperparameters within the following parameter grid:

- Neurons/memory unit: [32,64,128].
- Optimizer: [Adam, RMSprop].
- Learning rate: [0.001,0.01].
- Batch size: [8,16,64,128].
- Epochs: [20,30].

This method involves evaluating all the required configurations by entirely testing various combinations of hyperparameters using the cross-validation splitting strategy. To account for the stochastic nature of the learning algorithms, empirical experimentation was conducted by running the algorithms of interest ten times. In each case, the parameters that yielded the smallest RMSE were selected, considering both the mean value and the standard deviation. The best hyperparameter values found by grid search for MLP, LSTM Model 1, and LSTM Model 2 models are presented in Table 5.

### 4.5. Experimental Results and Analysis

The comparison of positioning results and statistics (min, max, std) for various deep learning methods, obtained by running them 10 times in individual and combined test areas, is presented in Table 6. The performance of the positioning algorithms differs across each experimental area due to changes in the environment as seen in Table 6.

**Figure 12:**
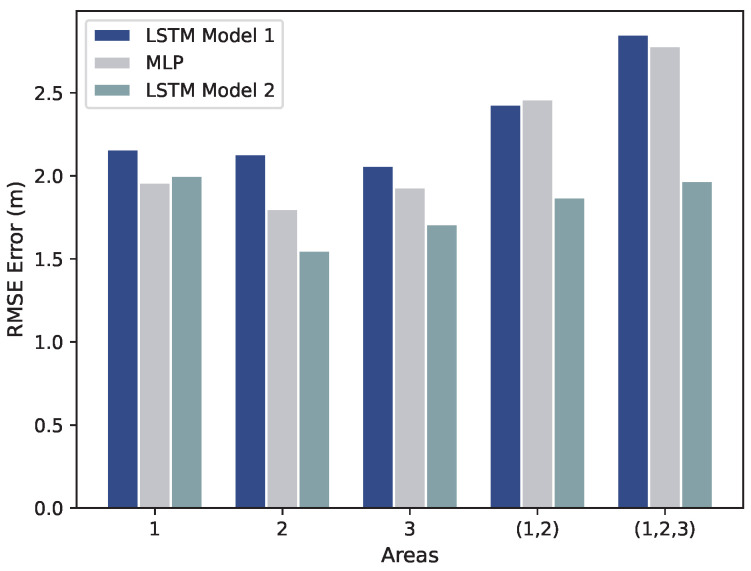
Positioning performance of used algorithms per different mission areas.

From Table 6, it can be concluded that the average performance degrades by up to 2.06 m in LSTM Model 1, 1.80 m in MLP, and 1.55 m in LSTM Model 2. LSTM is capable of capturing the correlated RSS measurements and positions over time. Additionally, it indicates that the inclusion of additional position information in LSTM Model 2 has a positive effect on location estimation. Based on these results, as shown in Figure 12, LSTM Model 2 outperforms MLP and LSTM Model 1 in all areas except Area 1, where MLP performs better. Furthermore, LSTM Model 2 also significantly improves positioning accuracy, particularly in combined areas, Area (1, 2, 3).

**Figure 13:**
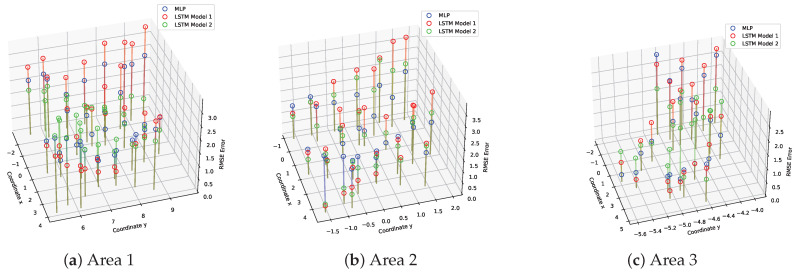
Comparison of the individual positioning errors in meters obtained from various test points across different mission areas (**a**–**c**). The comparison is made between three models: MLP, LSTM Model 1, and LSTM Model 2.

Figure 13 depicts the positioning RMSE for varying numbers of test points (39, 34, and 26) across Areas 1, 2, and 3, respectively, considering different algorithms. The errors are represented by the z entries, extending from the xy-plane, where x and y correspond to the locations of RPs in the xy-plane. To enhance clarity in the visualization, discrete test errors are overlaid on a 2D map of the environment using a color-coded (blue–green–red) style spectrum. A continuous, complete error map is generated by applying a radial basis Gaussian function interpolation method [62] with parameters ϵ=0.04 m and σ=0.01 m. Here, ϵ represents an adjustable constant for the Gaussian function, while σ controls the smoothness of the approximation.

**Figure 14:**
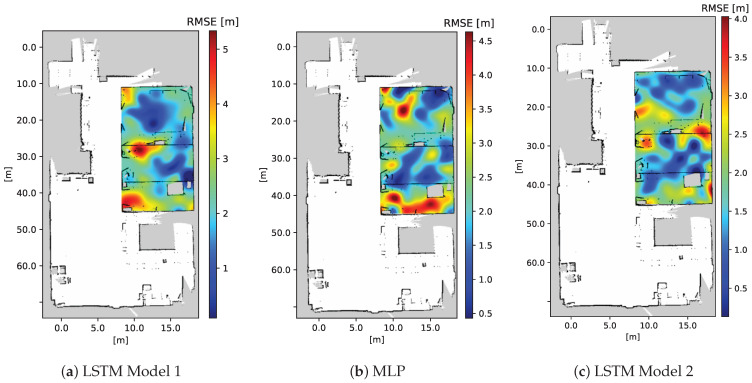
Positioning RMSE heat map in meters for each positioning algorithm used. In this heat map, lower RMSE values are indicated by the blue color, while higher RMSE values are represented by the red color.

Figure 14 displays the RMSE error heat map for each algorithm in the mission areas. In this representation, the blue color indicates lower RMSE values, while the red color indicates higher RMSE values. From Figure 14, it is shown that MLP and LSTM Model 1 have high RMSE in Area 3, while LSTM Model 2 achieves better results across Areas 1, 2, and 3. This improvement results from incorporating position information from the previous additional step within LSTM Model 2. Additionally, higher RMSE values are observed in areas with $90^{\circ }$ turns and boundary regions that separate areas with fences made of materials like metal, glass, and brenda. This is expected, as these areas and materials are more prone to error sources such as multipath propagation and shadowing.

**Figure 15:**
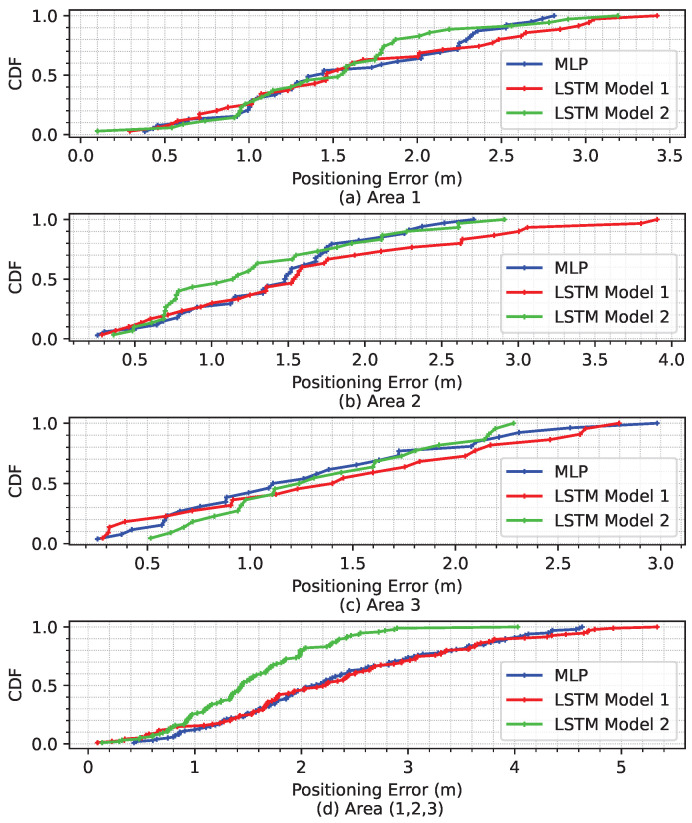
CDFs of individual positioning errors in all areas for all models.

The cumulative density function (CDF) of the positioning RMSE errors for the positioning algorithms can be seen for both individual and combined mission areas, as depicted in Figure 15. Moreover, as can be seen from Figure 15d, the accuracy of positioning is significantly improved by employing the LSTM Model 2 algorithm for positioning performance compared to the other algorithms used in the combined Area (1, 2, 3). In order to provide an intuitive demonstration, the CDF values within 2 m of the positioning error of the techniques used are also given in Table 7. The CDF value within 2 m of the MLP algorithm is 85.29% in Area 2, which is better than the other algorithms as can be seen in Table 7. On the other hand, LSTM Model 2 achieved the best results in all other areas, with values higher than 80%.

## 5. Discussion and Conclusions

In this paper, we present the architecture of a cyber-physical wireless indoor positioning system and its components, aligning with the five properties that define a cognitive digital twin compared to the literature. These properties, outlined in Section 3.1, include scalability, situational awareness, minimization of reliance on human presence, precise and efficient data collection, and an efficient communication network. The proposed cyber-physical indoor positioning system demonstrated achievements to fill in the gaps in the requirements for cognitive digital twins of indoor positioning systems, as can be seen from Table 8.

The integration of autonomous mobile data collectors guided by a digital twin showcased the adaptability and scalability of the fingerprinting-based systems for diverse indoor settings and challenging remote environments. The synchronized 3D digital twin environment and graphical user interface enhanced situational awareness, empowering human operators for informed decision-making. By successfully minimizing reliance on human presence through autonomous Wi-Fi site surveying, the system not only reduced labor costs but also improved safety. Moreover, the adoption of interactive reference point decision-making and the SLAM algorithm provided precise and efficient data collection, ultimately improving the accuracy of fingerprint-based positioning methods. The utilization of a defacto standard ROS framework for efficient communication further coordinated the system’s capabilities with its provision of standardized functionalities as an open-source middleware and flexible framework designed for the development of robot software that can easily scale and be interchanged.

The feasibility of the proposed CPS architecture is demonstrated by utilizing an autonomous robotic platform connected to a DT through a communication network that is coordinated by human-in-the-loop interaction. This approach enables remote operations and allows for regular re-adjusting or updating to effectively address the challenge of radio map obsolescence, aiming to mitigate the undesirable effects of time variations in RSS due to environmental conditions. It can also make remote operations safer and more efficient, particularly in environments where traditional fingerprinting systems may be ineffective or impractical, such as remote or hazardous locations. For instance, in nuclear facilities where human access is restricted or hazardous, the system can provide accurate positioning information without the need for personnel to physically enter the facility.

Furthermore, the proposed approach takes advantage of the good precision of deep learning models, resulting in localization performance comparable to that of prior studies [16,55]. Three deep neural network fingerprinting techniques have been developed for indoor WLAN-based positioning, using a radio map obtained from a real-world environment. Experimental results indicate that the LSTM Model 2 network architecture achieves the most accurate positioning performance in all areas except Area 1. Also, the MLP outperforms the LSTM Model 1 significantly in Area 2 and Area 3 with a CDF of more than 80% within a 2 m error, while the LSTM Model 2 achieved CDF values of more than 80% in all fields. This highlights the significance of incorporating additional position information in LSTM Model 2.

These results show the effectiveness of the proposed cyber-physical wireless indoor positioning approach, characterized by the application of dynamic Wi-Fi RSS surveying using autonomous mobile robots and the incorporation of human feedback through a graphical user interface. This shows its practical applicability and suitability for deployment in real-world scenarios. To improve positioning accuracy, additional information can be incorporated into the training of deep learning models. This information may include the receiver’s four orientations (north, south, east, and west), various signal characteristics (such as angle of arrival and time of arrival with multipath profile), and knowledge of the motion dynamics of the asset being tracked (e.g., human, robot, etc.).

Moreover, as a contingency plan for a potential issue of robot kidnapping, wherein the robot cannot estimate its location through SLAM to label the fingerprint location, a human-in-the-loop can engage in the process. They can take control to assist the robot in relocating itself through teleoperation, utilizing a graphical user interface through a digital twin. Once the robot is repositioned, it can resume its mission to collect the remaining fingerprint locations. In the absence of a human observer, the following kidnapping detection approaches [63,64] can be employed in SLAM to take action, such as defining recovery motions, especially in the case of a short-range kidnapping situation.

One limitation of the proposed CPS design is its potential inadequacy in addressing the resilience of the communication network and computing resource constraints in different environments. This can result in unexpected challenges, reliability issues, and performance problems during system implementation. To overcome these limitations, several strategies can be employed. Firstly, network design can be implemented to tolerate communication disruptions, enabling the system to connect and disconnect as needed. This approach ensures that the system can continue to function even in the event of temporary network interruptions. Secondly, prediction strategies can be employed to compensate for dropped or missed packets, thereby enhancing the system’s reliability. These strategies also allow the system to proactively adapt and mitigate the impact of communication disruptions. Furthermore, smart data management techniques, combined with data minimization approaches or computing at the edge, can be implemented to reduce bandwidth usage and optimize the system’s reliability within bandwidth and computing resource-constrained environments.

Moreover, further research by conducting long-distance experiments that utilize cloud or peer-to-peer network architectures can follow the current approach. Additionally, the a priori RSS heat maps generated from the radio map on DT can be leveraged to effectively deal with uncertainties in RSS measurements through proactive radio scene occupancy analysis. These informed channel planning decisions can lead to physical environment improvements in wireless coverage, such as the identification of wireless dead zones and interference sources, as well as areas with varying Wi-Fi signal strengths. These insights can then be combined with the adaptive determination of selecting RPs and APs used around a region of interest for an improvement in positioning error.

## Figures and Tables

**Table 2:** Various maturity levels of DT follow hierarchical naming conventions adapted from [46] for indoor positioning systems. "+" refers to inclusion, while "-" denotes exclusion.

Ref. ID	Data Twin	Asset Twin	Predictive Twin	Interactive Twin	Cognitive Twin
[6]	+	-	-	-	-
[48]	+	+	-	-	-
[2]	+	+	-	-	-
[5]	+	+	-	-	-
[49]	+	+	+	-	-
[1]	+	+	+	-	-
[35]	+	+	+	+	-
Proposed	+	+	+	+	+

**Table 3:** Approximate max. total bandwidth requirements.

Assets	Bandwdith Consumption (MB/s)	Network Speed (Mbps)
Scout 2.0 (pose, joint states, LIDAR)	4.546	36.368
2D Map (occupancy grid)	0.709	5.672
Wi-Fi scan (RSS measurements)	0.00068	0.00544
RP generator	0.026	0.208
Total	5.281	42.253

**Table 4:** Description of the number of reference points and APs for both train and test points across different areas, along with the reference point planner parameters.

		# Markers	# Inner loops	# RPs	# APs
Area 1	Train	4	3	70	17
	Test	6	1	39	17
Area 2	Train	4	2	41	19
	Test	6	1	34	19
Area 3	Train	4	1	25	18
	Test	6	1	26	18

# refers to the “number of”.

**Table 5:** MLP, LSTM Model 1, and Model 2 hyperparameters and cross-validated grid search results.

Hyperparameters	MLP	LSTM Model 1	LSTM Model 2
lag_size	-	4	4
hidden_layer	3	1	1
memory_unit	32	128	128
learning_rate	0.01	0.01	0.001
batch_size	16	8	128
optimizer	Adam	RMSprop	RMSprop
epochs	30	20	20

**Table 6:** The positioning RMSE performance statistics were obtained by running each technique of interest (the LSTM Model 1, MLP, and LSTM Model 2) ten times. The results are reported in meters.

Area	LSTM Model 1					MLP					LSTM Model 2			
	Average	Min	Max	Std		Average	Min	Max	Std		Average	Min	Max	Std
1	2.16	2.12	2.22	0.03		1.96	1.88	2.08	0.05		2.00	1.39	2.37	0.26
2	2.13	1.94	2.52	0.15		1.80	1.72	1.84	0.03		1.55	1.11	2.08	0.31
3	2.06	1.96	2.29	0.09		1.93	1.75	2.48	0.22		1.71	0.95	2.77	0.61
(1, 2)	2.43	2.03	2.81	0.26		2.46	2.22	2.74	0.18		1.87	1.32	2.81	0.42
(1, 2, 3)	2.85	2.66	3.23	0.17		2.78	2.56	3.06	0.15		1.97	1.60	2.47	0.25

The values are given in meters (m).

**Table 7:** The probabilities of positioning errors for all models up to within 0–2 m in all areas.

Positioning Alg.	CDF Value (%)			
	Area 1	Area 2	Area 3	Area (1, 2, 3)
MLP	64.10	85.29	80.76	46.46
LSTM Model 1	65.71	73.33	72.72	46.31
LSTM Model 2	82.85	83.33	86.36	81.05

**Table 8:** Assessment of findings: Problem Statements vs. Experimental Results.

Problem Statements	Assessments of Results
1. **Scalability:** The system should be adaptable and applicable to diverse indoor settings as well as challenging remote environments.	1. The utilization of autonomous mobile data collectors guided by a digital twin demonstrates the potential for scaling up the system in constructing radio maps for fingerprinting-based methods. While a single mobile robot is considered, the approach is adaptable to multi-robot fleets, making it suitable for large indoor spaces with multiple floors.
2. **Situational Awareness:** The system should augment situational awareness and support humans in making better decisions.	2. The system provides a synchronized 3D digital twin environment and a graphical user interface to enhance human collaboration, contributing to an improved understanding of indoor environments.
3. **Minimize the reliance on human presence:** The system should minimize human presence in the field to both lower the costs of labor related to data collection and improve the safety of personnel and facilities.	3. The system has reduced labor costs associated with data collection by replacing manual efforts with autonomous Wi-Fi site surveying. This minimizes the manual effort required for data gathering and maintenance, consequently improving human safety in hazardous environments.
4. **Precise and efficient data collection:** The system should provide precise ground-truth data collection for more accurate positioning methods.	4. Utilizing interactive reference point decision-making and SLAM algorithm for Wi-Fi site surveys resulted in more efficient and precise data collection.
5. **Efficient communication network:** The system should provide acceptable latency in updates to provide timely and actionable information for seamlessly integrating physical and digital spaces.	5. A de facto standard ROS framework is used for digitalization and software tools. It helps to coordinate a cyber-physical indoor positioning ecosystem with its distributed software communication architectures.

## Data Availability

The data presented in this study are available on request from the corresponding author. The data are not publicly available due to privacy.
